# Multi-omics knowledge spectrum of osteoporosis: A bibliometric and visual analysis

**DOI:** 10.1097/MD.0000000000044886

**Published:** 2025-10-03

**Authors:** Yuxia Yang, Xuyong Gong, Tao Bao

**Affiliations:** a School of Medicine, Yangzhou University, Yangzhou, PR China; b Department of Orthopedics, Haimen People’s Hospital, Nantong, PR China; c Department of Orthopedics, Chun’an First People’s Hospital, Hangzhou, PR China.

**Keywords:** bibliometrics, metabolomics, multi-omics, osteoporosis, proteomics, transcriptomics

## Abstract

**Background:**

Osteoporosis is a systemic condition that often goes unnoticed, marked by a reduction in bone density and mass, deterioration of bone microstructure, and heightened susceptibility to fractures. In recent years, numerous scientists have conducted large-scale omic studies on osteoporosis; however, there is no systematic bibliometric and visualization analysis in this area.

**Methods:**

In the present investigation, literature concerning omic research on osteoporosis from the early 21st century was retrieved from the primary database of the Web of Science. Subsequently, the collected data underwent statistical and visual analysis utilizing tools such as CiteSpace, VosViewer, and R.

**Results:**

In this investigation, a total of 1148 scholarly articles were gathered, revealing a consistent annual increase in publication numbers. The preceding 5 years have marked a significant phase of advancement in the field of osteoporosis omics research. Historically, the United States has maintained a dominant position in this domain until 2014; however, several Asian nations have experienced swift progress and noteworthy breakthroughs over the past 10 years. The application of omic techniques within the field of osteoporosis has evolved at a phenomenal rate, going through 3 major phases. The first phase of research focused on omic studies on a large number of mixed cells; the second phase delved into gene expression studies to the single-cell level, which in turn led to in-depth characterization of cell types and revealed cellular heterogeneity; and the third phase progressively carried out in-depth studies on the acquisition of gene expression profiles and spatial distribution data from tissues in situ.

**Conclusions:**

This study represents the inaugural bibliometric and visualization examination of published research findings pertaining to osteoporosis, achieved through a systematic data search and the integration of various bibliometric analysis instruments. Utilizing these data, we synthesize prior scholarly investigations and offer a perspective on forthcoming research directions within this domain.

## 1. Introduction

Osteoporosis is a systemic disease characterized by a decrease in bone density and quality, damage to bone microstructure, and an increase in bone fragility, and has been called “the silent disease.”^[1]^ Osteoporosis is a major public health disease worldwide, affecting the lives and health of countless people and resulting in a huge economic burden.^[2]^ Osteoporosis may not cause any obvious discomfort in the early stages, but as the disease progresses, it often manifests itself in pain, spinal deformity and fragility fracture.^[3]^ These symptoms can lead to pain, dysfunction in daily life, or more serious complications such as thoracic deformities that affect cardiopulmonary function.

The pathophysiologic mechanisms of osteoporosis are complex, and the most common are postmenopausal osteoporosis and senile osteoporosis. Postmenopausal osteoporosis is mainly caused by the cessation of ovarian function during menopause, resulting in a significant decrease in estrogen levels, and its onset is common in women.^[4]^ Unlike postmenopausal osteoporosis, senile osteoporosis is mainly due to aging and therefore affects both men and women.^[5]^ Although there are different types of osteoporosis, some of the major common pathologic changes are the dysregulation between osteoclast-activated bone production and osteoblast-activated bone destruction and the loss of compensation for cellular senescence.^[6]^ Therefore low bone mineral density is one of the key features of osteoporosis and one of the main criteria for diagnosis at present.^[7]^ Despite significant advances in pharmacologic interventions in the therapeutic strategies for osteoporosis, 8 however, more attention is currently focused on symptomatic treatment of osteoporosis, and there are no effective preventive measures for people at high risk for osteoporosis.^[8]^ The search for important targets in the pathogenesis of osteoporosis remains a future endeavor for the development of effective prevention strategies.

Exploring the mechanisms of disease onset and progression is an important step in clinical translation, and the initial mapping of the human genome by 6 countries in 2000 and the significant progress made by the “Moon Landing Project” research projects in the life sciences provide avenues for studying the mechanisms of disease onset and progression.^[9,10]^ Since then, there has been an emergence of omic technologies such as transcriptomics,^[11]^ proteomics and metabolomics.^[12]^ In recent years, in addition, lipidomics, epigenomics, and single-cell omic have emerged with the continuous development of sequencing technologies. Each of these omic technologies may contribute to the discovery of key mechanisms of osteoporosis at different levels, however this is not comprehensive enough. Previous traditional gene expression studies have suggested that osteoporosis has a complex ontogeny controlled by multiple intracellular mechanisms, such as regulation at the transcriptional level, modification at the protein level, and other metabolic levels,^[13]^ and a comprehensive analysis by utilizing a systems biology viewpoint and combining multiple omic data is necessary.

In 1934, the term bibliometrics was established as a systematic field that employs mathematical and statistical methodologies to quantitatively assess published literature. This approach serves as an efficient method for promptly acquiring an overview of the current research landscape within a particular domain.^[14,15]^ The most commonly used scientific method in bibliometrics is citation analysis, which is valuable for identifying current hotspots and discovering key issues by analyzing previous studies,^[16,17]^ which is also very different from similar previous reviews.^[18]^ In addition, bibliometric analysis has been widely applied to a variety of diseases within medicine, such as cardiology,^[19]^ obstetrics and gynecology,^[20]^ and urology,^[21]^ where researchers can answer questions in the field based on publication data (e.g., institutions, authors, keywords) in the same way that epidemiologists learn about the health of a population based on querying patient data.^[22]^

The swift advancement of omic technologies in recent years has catalyzed a transformative change towards the precise treatment of diseases.^[10,23,24]^ From our previous review of the research field, we found that some scholars have only conducted relevant analyses of high-impact studies^[25]^ and treatment strategies within osteoporosis,^[26–28]^ and many scholars may lack a holistic view of omic research within the field of osteoporosis. Therefore, this study aims to comprehensively collect articles related to osteoporosis omic research since 2000, summarize and visualize the results, identify changes in research hotspots, assess countries, institutions, journals and authors with significant contributions, and discover high-quality journals in the field. Our work helps subsequent researchers to quickly learn the current research status in the field and guides researchers in the direction of external academic exchanges and research collaborations.

## 2. Materials and methods

### 2.1. Data sources and search strategies

This study does not involve ethical issues. The articles included in this study were derived from the Web of Science Core Collection (WOSCC) database, which is the most commonly used database for bibliometric studies and ensures a comprehensive citation record, while the WOSCC ensures a high quality of the literature. We systematically searched for articles published between January 1, 2000 and January 1, 2025, limiting the type of literature to treatises and reviews, excluding literature in languages other than English, and with no restriction on nationality or specialty. The search strategy is: TS=(transcriptomic OR proteome OR proteomic OR metabolomics OR lipidomics OR metagenomics OR metatranscriptomics OR omics OR microarray OR RNA-seq OR sequencing OR ATAC-seq OR “single cell sequencing” OR “single-cell omics” OR “single cell sequence” OR “single cell RNA sequencing” OR “single cell RNA sequence” OR “expression profile” OR epigenomics OR bioinformatic* OR high throughput OR mass spectrometry OR “genome-wide association studies” OR GWAS) AND TI= (osteoporosis OR osteoporoses OR oeteoporotic OR osteopenia OR “low bone density” OR “low bone mass”). We screened the retrieved literatures one by one through abstracts, and excluded the literatures whose contents were not related to osteoporosis-related research. This work was carried out by 2 researchers at the same time. Finally, the results retrieved by the 2 researchers were summarized, and the repeated or irrelevant literatures were screened again. We exported all search results from Web of Science (WOS) in plain text format and stored them temporarily for ready access and further in-depth analysis.

### 2.2. Data collection

To mitigate potential data inconsistencies arising from updates to the database and other factors, data was meticulously screened and downloaded on January 1, 2025. The bibliometrix package version 4.0.1, which is widely utilized for the collation and visualization of bibliometric data, was installed within R software for thorough data verification and cleansing. Subsequently, key details, including article titles, journal names, publication years, countries, institutions, authors, and keywords, were extracted and saved into Microsoft Excel. The plain text files obtained were also imported into R for the purpose of data validation and cleaning, leading to the exclusion of literature not pertinent to the current study. Important metadata such as article title, journal, year, country, institution, author, and keywords were then organized and stored in Microsoft Office Excel (version 2019).

### 2.3. Bibliometric analysis

Scientific visualization and analysis provides a more intuitive and clearer presentation of progress and trends within the research field, and this study combined a variety of software, including R software (Version 4.2.1), Excel (Version 2019), Citespace (Version 6.3.R2Advanced), VOSviewer (Version 1.6.19) and SCImago Graphica (Version 1.0.42), and the data visualization process followed the instructions for using the software. With R software, specific information such as authors, institutions and countries can be effectively visualized, providing rich data illustrations and analysis results.^[29]^ Excel possesses the capability to effectively quantify the yearly publication rates of articles, aggregate citation counts, and compute the mean citation values. Additionally, it offers visualization tools that facilitate researchers in interpreting and analyzing trends within the data with ease. Citespace conducts an analysis of potential connections within scientific literature, elucidating research hotspots and the progression of knowledge over time. This tool offers a comprehensive overview of the knowledge structure, thereby assisting researchers in comprehending the interrelationships among various literature and the evolution of the academic network. Citespace provides a clear knowledge structure mapping to help researchers understand the connection and development of the literature.^[30]^ VOSviewer is able to build and view complex bibliometric networks, revealing trends and interrelationships in different research areas through analysis and visual mapping.^[31]^ SCImago Graphica, on the other hand, provides a global perspective on scientific research collaboration and international cooperation by mapping the world and constructing partnerships between countries, providing researchers with a comprehensive picture of international cooperation.^[32]^ Using a combination of these tools, bibliometric studies allow for more comprehensive and in-depth analyses that reveal the dynamics and trends of scientific research.

## 3. Results

### 3.1. Overview of omic research on osteoporosis

**Figure 1.**
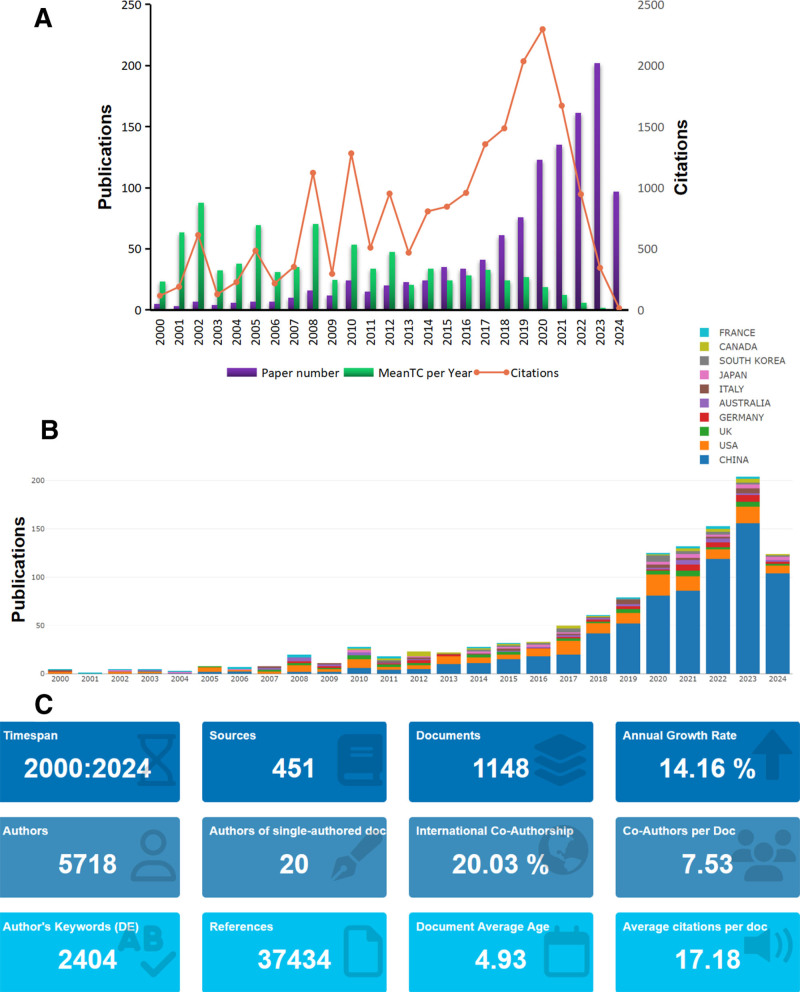
General overview of published papers. (A) Year distribution, number of citations, and average number of citations for osteoporosis omic studies. (B) Country distribution of the number of published papers. (C) General information of osteoporosis omic studies.

Utilizing the search strategy outlined in the Methods section, we successfully identified a total of 1148 qualifying articles (Fig. 1A). Notably, the volume of articles exhibited a year-on-year increase, revealing 3 distinct phases. The initial phase spans from 2000 to 2014, characterized by a gradual increase in the volume of published articles; the second phase is from 2015 to 2019, a time period in which the growth of articles increases faster; and the third phase is from 2020 to 2024, a 5-year period in which the number of published articles grows extremely fast and the number of articles exceeds 100/yr. It can be noticed that the United States has dominated the field from 2000 to 2013, while China has issued a very high percentage of articles in the last decade (Fig. 1B). A total of 1148 articles were disseminated across 451 journals, with contributions from 5718 authors globally, referencing an aggregate of 37,434 sources (Fig. 1C).

Based on the statistics from the WOS presented in Table 1, the 3 most frequently cited publications are as follows: The foremost article is authored by Kemp, JP, titled “Identification of 153 new loci associated with heel bone mineral density and functional involvement of GPC6 in osteoporosis,” which was published in 2017 in the journal NATURE GENETICS and has garnered a total of 302 citations. Following closely is the second article, “An algorithm recommendation for the management of knee osteoarthritis in Europe and internationally: A report from a task force of the European Society for Clinical and Economic Aspects of Osteoporosis and Osteoarthritis (ESCEO),” authored by Bruyère, O, and published in 2014 in SEMINARS IN ARTHRITIS AND RHEUMATISM, which has received 293 citations. Lastly, the third article, “Genetics of Osteoporosis,” by Ralston, SH, published in the journal ENDOCRINE REVIEWS in 2010, has been cited 258 times.

**Table 1:** The 10 most-cited papers.

Ranking	Title	Journal	Author	Year	Citations
1	Identification of 153 new loci associated with heel bone mineral density and functional involvement of GPC6 in osteoporosis	Nature Genetics	Kemp, JP	2017	302
2	An algorithm recommendation for the management of knee osteoarthritis in Europe and internationally: a report from a task force of the European Society for Clinical and Economic Aspects of Osteoporosis and Osteoarthritis (ESCEO)	Seminars in Arthritis and Rheumatism	Bruyère, O	2014	293
3	Genetics of osteoporosis	Endocrine Reviews	Ralston, SH	2010	258
4	Nephrolithiasis and osteoporosis associated with hypophosphatemia caused by mutations in the type 2a sodium–phosphate cotransporter	New England Journal of Medicine	Prié, D	2002	253
5	Disease mechanisms genetics of osteoporosis from genome-wide association studies: advances and challenges	Nature Reviews Genetics	Richards, JB	2012	213
6	Endogenous sex hormones and incident fracture risk in older men – the dubbo osteoporosis epidemiology study	Archives of Internal Medicine	Meier, C	2008	201
7	LncRNA MALAT1 shuttled by bone marrow-derived mesenchymal stem cells-secreted exosomes alleviates osteoporosis through mediating microRNA-34c/SATB2 axis	Aging-Us	Yang, XC	2019	190
8	A look behind the scenes: the risk and pathogenesis of primary osteoporosis	Nature Reviews Rheumatology	Hendrickx, G	2015	190
9	A road map for understanding molecular and genetic determinants of osteoporosis	Nature Reviews Endocrinology	Yang, TL	2020	189
10	Notch inhibits osteoblast differentiation and causes osteopenia	Endocrinology	Zanotti, S	2008	166

### 3.2. Visualization and analysis of keywords

**Figure 2.**
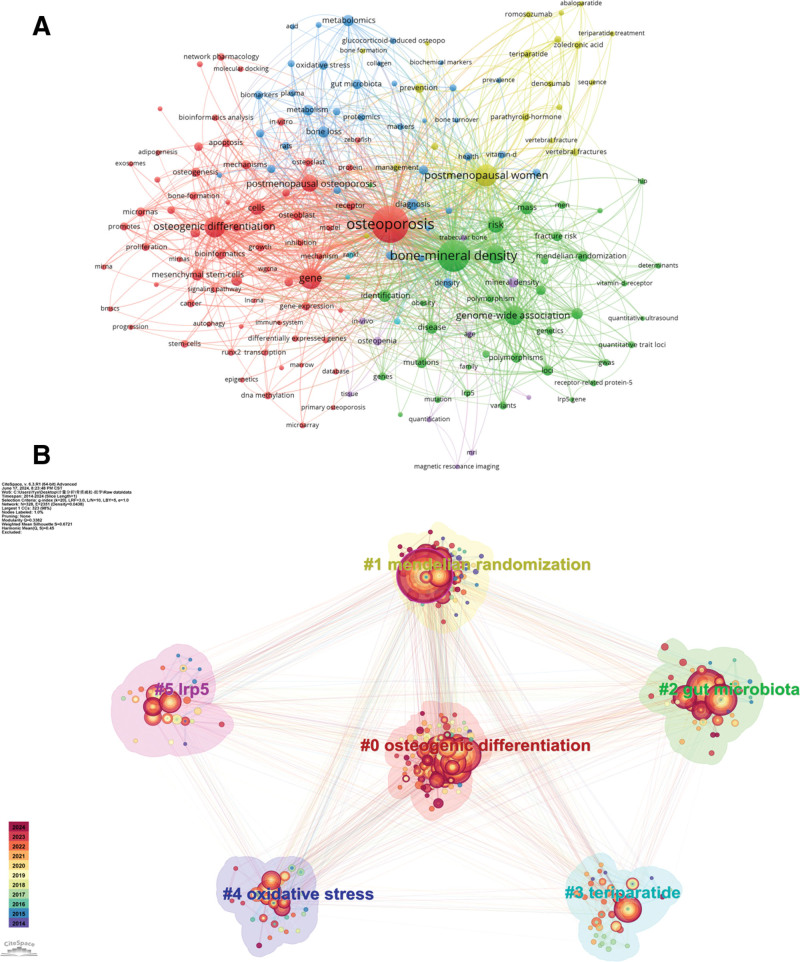
Keyword characterization of an omic study of osteoporosis. (A) Keyword visualization mapping, each node represents a keyword. (B) Keyword clustering situation, different colors represent different clusters.

Keywords serve to encapsulate the core concepts of an article, thereby enabling the identification of current hotspots and emerging trends within the realm of osteoporosis omic research. Utilizing VOSviewer, we created a visual representation of these keywords. In this visualization, the dimensions of the circles corresponding to each keyword are directly proportional to their frequency of occurrence. Additionally, the lines connecting pairs of keywords indicate their co-occurrence within the same publication. It can be found that the 5 most frequently used keywords in the omic studies of osteoporosis are “osteoporosis,” “bone-mineral density,” “ postmenopausal women,” “osteogenic differentiation”, and “gene” (Fig. 2A, Table 2). The keywords “osteoporosis” and “bone-mineral density” were in the center of the clusters of different studies.

**Table 2:** Top-20 keywords with the highest frequency.

Ranking	Keyword	Frequency	Ranking	Keyword	Frequency
1	osteoporosis	658	11	mass	69
2	bone-mineral density	469	12	identification	63
3	postmenopausal women	218	13	disease	58
4	osteogenic differentiation	201	14	mesenchymal stem-cells	56
5	gene	196	15	diagnosis	55
6	genome-wide association	162	16	Meta analysis	54
7	fracture	153	17	bone loss	52
8	postmenopausal osteoporosis	120	18	metabolomics	52
9	risk	113	19	osteoblast differentiation	50
10	cells	90	20	mendelian randomization	48

Concurrently, we conducted a comprehensive cluster analysis of the keywords, resulting in the identification of 6 primary research directions: #0osteogenic differentiation, #1mendelian randomization, #2gut microbiota, #3teriparatide, #4oxidative, #5Irp5, and the keywords were categorized into related clusters (Fig. 2B, Table 3). It can be observed that the clusters contain roughly the same range of keywords, suggesting that the number of osteoporosis omic studies is relatively even in all directions.

**Table 3:** Information about all keyword clusters.

Cluster ID	Label	Size	Silhouette	Mean (yr)
0	Osteogenic differentiation	103	0.548	2018
1	Mendelian randomization	83	0.699	2018
2	Gut microbiota	43	0.707	2019
3	Teriparatide	36	0.84	2018
4	Oxidative stress	30	0.811	2017
5	Irp5	28	0.634	2017

**Figure 3.**
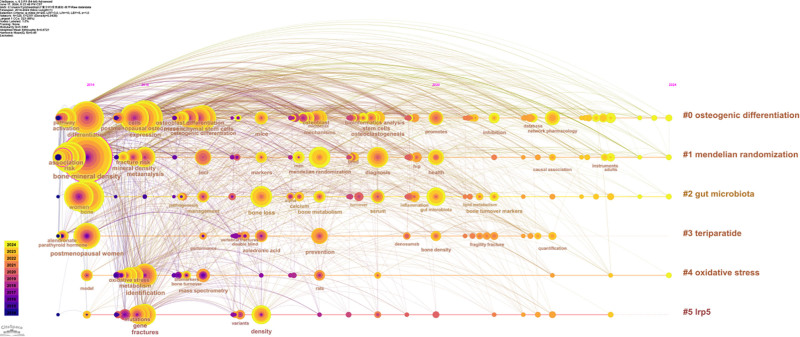
Evolution of keywords in the osteoporosis omic study from 2000 to 2024. Larger circles suggest a higher frequency of occurrence, and the color of the chronology represents the time of occurrence.

The timeline graph illustrating keywords (Fig. 3) depicts the evolving trends associated with these terms. In this representation, the size of each node correlates with the frequency of its most recent appearance, while the horizontal positioning of the nodes indicates the timing of their initial emergence. Furthermore, the color gradient of each node’s annulus transitions from purple to red, reflecting the average time of occurrence, with the shift in hue corresponding to an increase in chronological order. The #0osteogenic differentiation cluster contains the richest evolution of keywords and is more often associated with keywords from other clusters. It can be found that the keywords “differentiation,” “bone mineral density”, and “expression” are longtime research hotspots. In addition, some new hotspot keywords appeared late in recent years, such as “gut microbiota,” “network pharmacology,” “osteoclastogenesis”, and “causal association,” which indicate new research directions in this field.

### 3.3. Analysis of references

**Figure 4.**
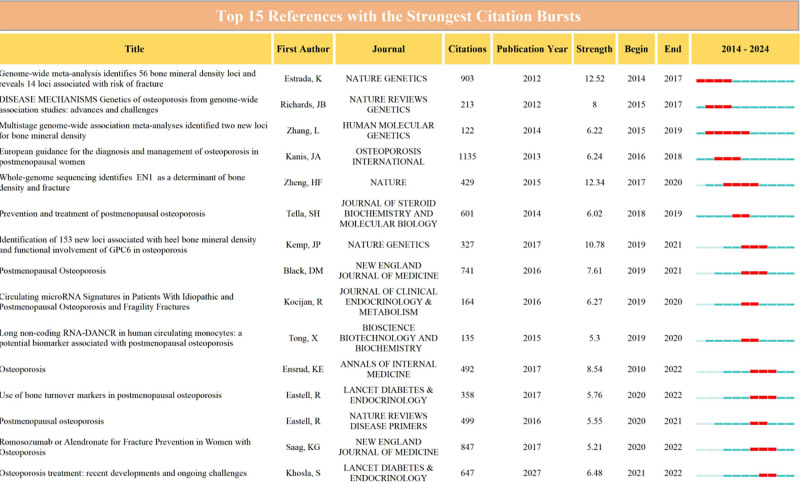
The 15 most relevant cited references.

The references included in a scholarly article can indicate the research trajectory of the literature, as evidenced by the citation of 37,434 references in total, highlighting a robust foundational research base within the current domain of inquiry (Fig. 1C). Furthermore, Figure 4 illustrates the 15 references that experienced the most significant citation surges, with the blue line denoting the timeframe during which the cited literature emerged, and the red line indicating the period of heightened citation frequency. One of the longest lasting outbreaks was the 2014 publication in *HUMAN MOLECULAR GENETICS* of “Multistage genome-wide association meta-analyses identifying 2 new loci for bone mineral density.”

### 3.4. Journal analysis

**Figure 5.**
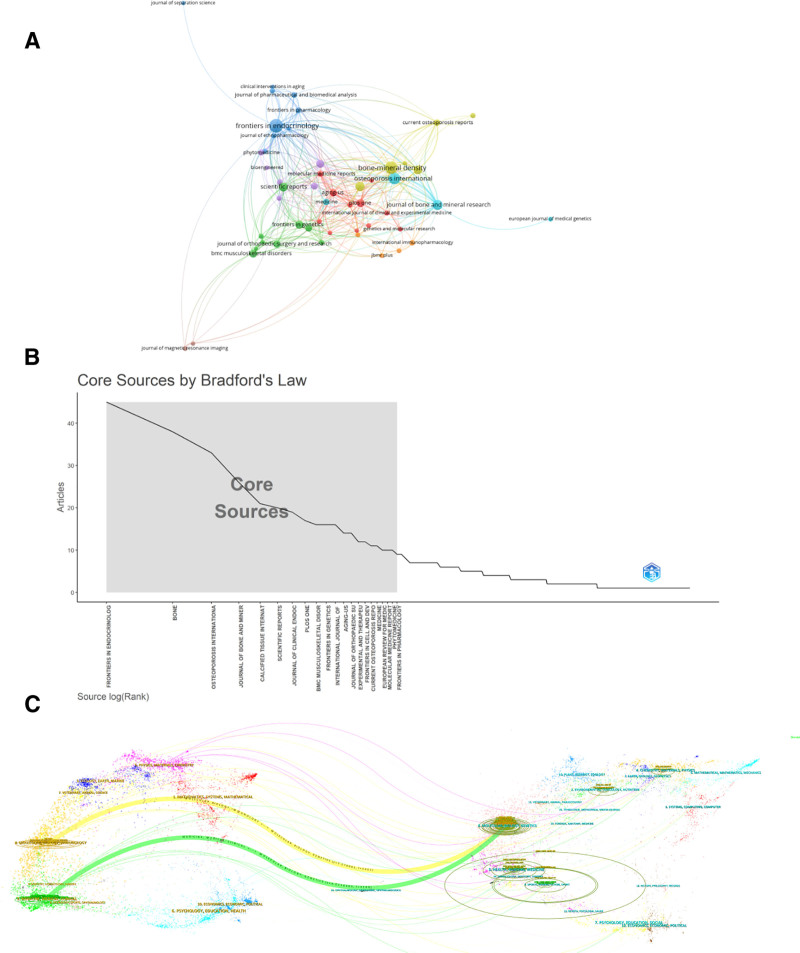
Journal analysis. (A) Visualization map of journals with published articles. (B) List of core journals calculated by Bradford’s law. (C) Double-stacked map of journals.

In the field of osteoporosis omic research, we calculated the 10 most relevant published journals using the R package (Fig. 5A, Table 4). Some of the special journals in the endocrine and osteoporosis direction made a large contribution, with *Frontiers In Endocrinology* publishing the largest number of articles with 45, followed by *Bone-Mineral Density* with 38, and most of the others being general interest journals within the biomedical field. A total of 21 journals were categorized as core journals according to Bradford’s law (Fig. 5B). Furthermore, we employed a journal overlay map to illustrate the relationships between journals that cite and those that are cited (Fig. 5C). This representation indicates that the journals positioned on the left side reference the journals located on the right, with the connections denoting the relationships among these publications. The map predominantly reveals that research articles within the realms of molecular biology, immunology, medicine, and clinical medicine are mainly referenced by articles pertaining to molecular biology and genetics within the context of osteoporosis omics research.

**Table 4:** Top-10 journals ranked by the number of publications.

Ranking	Journals	Publications	Citations	Average (citations/publications)
1	Frontiers in Endocrinology	45	420	9.33
2	Bone-Mineral Density	38	906	23.84
3	Osteoporosis International	33	512	15.52
4	Journal of Bone and Mineral Research	26	922	35.46
5	Calcified Tissue International	21	251	11.95
6	Scientific Reports	20	314	15.70
7	Journal of Clinical Endocrinology and Metabolism	19	475	25.00
8	Plos One	17	740	43.53
9	BMC Musculoskeletal Disorders	16	106	6.63
10	Frontiers in Genetics	16	95	5.94

### 3.5. Country analysis

**Figure 6.**
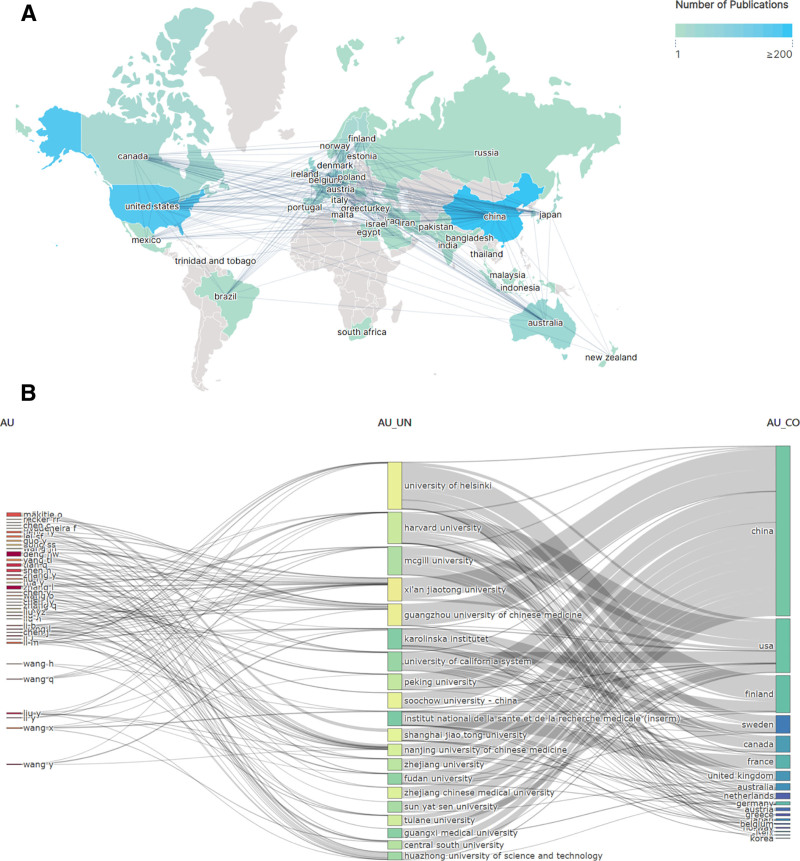
Country analysis of osteoporosis omic studies. (A) National distribution of publications. (B) Top 20 international collaborative links.

We visualized and analyzed the countries that contributed, and the statistics included the countries of all contributing authors, which were mainly located in Europe and the Americas, with China having the highest number of articles (740), followed by the United States (175) and the United Kingdom (49; Fig. 6A, Table 5). The countries with the highest number of citations were China (8873), the United States (5619), and the United Kingdom (2252; Fig. 6B) illustrates the global partnerships among the leading 20 authors, institutions, and nations, highlighting that the majority of these collaborations are associated with China, alongside significant partnerships with the United States and various European nations.

**Table 5:** Top-10 countries ranked by the number of publications.

Ranking	Country	Publications	Citations	Average (citations/publications)
1	China	740	8873	11.99
2	United States	175	5619	32.11
3	United Kingdom	49	2252	45.96
4	Canada	29	1610	55.52
5	France	26	1525	58.65
6	Switzerland	16	1372	85.75
7	Australia	37	1280	34.59
8	Netherlands	20	1105	55.25
9	Germany	39	997	25.56
10	Italy	32	940	29.38

### 3.6. Organizational analysis

**Figure 7.**
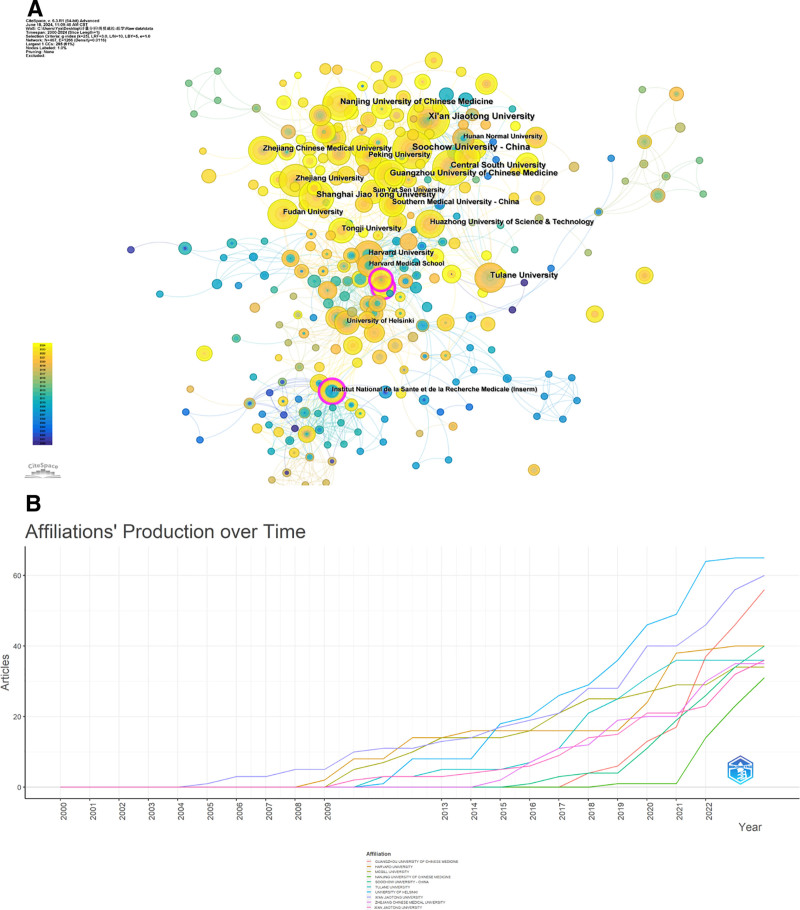
Institutional analysis. (A) Visual map of institutional collaboration. (B) Trends in postings for the 10 most relevant institutions.

In total, 1863 institutions participated in global contributions as indicated by the data. Utilizing Citespace software, we developed a visual representation of interinstitutional collaborations, where the connecting lines illustrate the cooperative relationships among the organizations. The dimensions or density of the circles correspond to the volume of citations received (Fig. 7A). Four institutions, Harvard University, the University of California, the French National Institute of Health and Medical Research, and the University of London, were at the center of the visualized network of collaborations, and these institutions had global collaborations, with fewer transnational collaborations at the other institutions, and a stable pattern of group collaborative research between more institutions (Fig. 6C). Figure 7B shows the publication trends of the top 5 institutions involved in the volume of publications. Among them, Harvard University was involved in the largest number of publications, followed by Nantong University and the University of London. 2012 was a turning point, and these institutions saw a sharp rise in the number of papers they were involved in after 2012.

### 3.7. Author’s analysis

**Figure 8.**
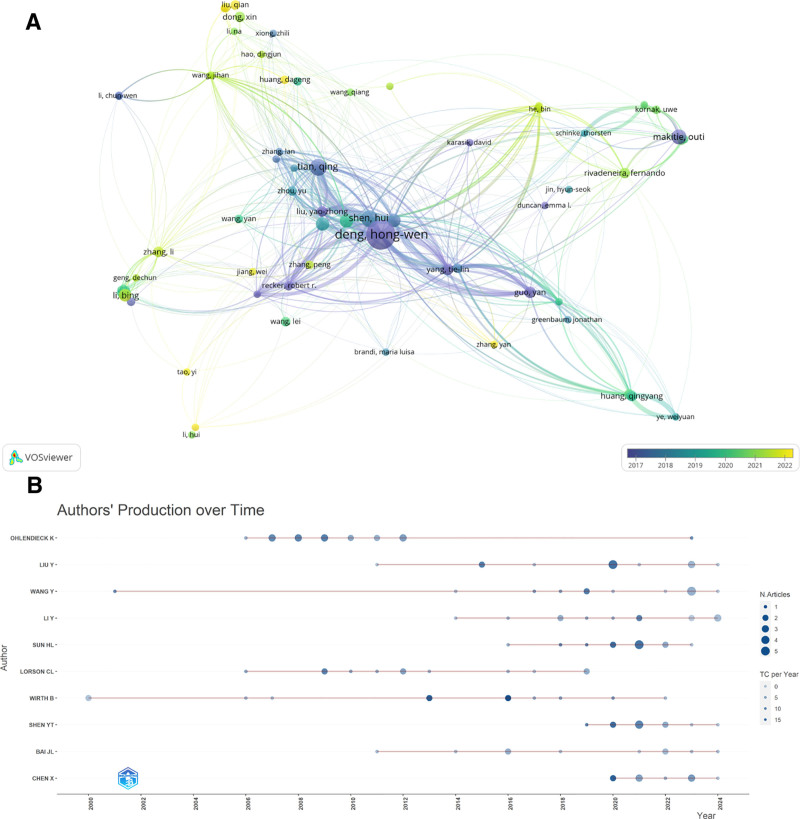
Author analysis. (A) Visual map of author collaborations. (B) Trends in postings by the 10 most relevant authors.

A comprehensive total of 5718 authors participated in the contribution through data calculation. Figure 8A illustrates the collaborative network map of authors exhibiting the most significant partnerships. The dimensions of the circles correspond to the number of published works, while the thickness of the connecting lines reflects the level of collaboration. The analysis reveals that the primary collaborative efforts among authors predominantly revolve around scholars Hong-wen Deng, Qing Tian, and Tie-Lin Yang. These individuals tend to focus their research collaborations within their respective institutions, although there is some involvement from authors across multiple countries (Fig. 8B) lists the publication status of the top 10 authors who were involved in the number of articles published, and these authors were not only the first and corresponding authors, but most of them were involved in the publication of 5 or more papers, which contributed greatly to the study of osteoporosis omic.

## 4. Discussion

### 4.1. An overview of omic studies of osteoporosis

More than 20 years ago, the success of the Human Genome Project enabled humans to obtain, for the first time, a complete picture of the structure and sequence of their entire genome, which enabled a large number of genetic studies to be conducted with a “navigator.”^[33,34]^ Moreover, the strategies, ideas and technologies established by the Genome Project in the study of human beings have given rise to the concept of “precision medicine” and the clinical practice of personalized medicine. Today, omic has entered a “mature phase.” The cost of sequencing a human genome has dropped from more than 3 billion dollars 20 years ago to 100$ today, and the time required has ranged from several years to 1 day. However, it is difficult to popularize “precision medicine” only by genomics. Precision medicine is essentially to analyze, identify, validate, and apply biomarkers to large samples of people and diseases through multi-omics technologies with genomes as the core, so as to accurately find out the causes of diseases and the targets of treatments, and to realize personalized and precise treatments and improve the benefits of diagnosis, treatment and prevention, and thus improve the effectiveness of diagnosis, treatment and prevention of diseases diagnosis and treatment and prevention, so the arrival of the era of multi-omics is inevitable.

Esteemed journals play a pivotal role in the swift propagation of significant findings within the scientific domain. The majority of the articles identified in this review were disseminated through medical and biological journals. Additionally, scholars exhibit a preference for publishing their findings in specialized journals such as *Frontiers in Endocrinology and Bone-Mineral Density*, which focus on osteoporosis research. This analysis utilized Bradford’s law to identify 38 core journals relevant to the study. These identified journals serve as a valuable resource for guiding the academic endeavors of researchers engaged in osteoporosis-related omics, thereby enhancing the efficiency of literature information services and the utilization of information, as well as contributing to the scientific evaluation processes in this field.

Global partnerships have the potential to significantly accelerate advancements in research. The country distribution map clearly illustrates that China serves as both a key contributor to the omics research related to osteoporosis and a hub for international collaborations (Fig. 6A, B). It is evident that the majority of collaborative efforts are predominantly focused on developed nations, exhibiting strong partnerships primarily with China and the United States, while collaborations with other countries remain comparatively weak. We posit that significant regional disparities exist in the omics research pertaining to osteoporosis, largely due to China’s status as the nation with the highest prevalence of osteoporosis, representing nearly 50% of the global population of over 200 million individuals affected by this condition. Moreover, the economic and scientific research capabilities of individual nations constitute a significant determinant, with countries exhibiting advanced levels in these areas tending to exert greater dominance and influence within their respective domains. Historically, prior to 2013, the United States consistently held the top position regarding the volume of research conducted in this area, a fact that underscores the nation’s technological and scientific prowess. However, from 2014 onwards, China surpassed the United States in the quantity of published articles, indicating a notable shift in the landscape of research output. Additionally, the recent alterations in the contribution proportions of national literature over the past few years highlight a growing significance of bibliometric analysis in understanding these trends. The change in the proportion of national literature contribution in recent years suggests the importance of this bibliometric analysis, and highlights the rapid progress and significant leap of some Asian countries in this field.

Given that the quantity of published articles fails to accurately represent the research quality in a nation, we assessed the mean citation count within the country’s academic literature. The countries with the highest average number of citations are Switzerland (85.75), France (58.65), and Canada (55.52), a result that suggests that these 3 countries have contributed some valuable research in this field, a disparity that emphasizes the value of the indicator of the number of citations of statistical publications. We believe that global scientific and technological advances and increased transnational exchanges and cooperation will provide more valuable opportunities for less developed regions.

Given that China holds the highest share of published research articles, the majority of these publications are affiliated with Chinese institutions. The landscape of institutional collaborations predominantly revolves around Soochow University, Xi’an Jiaotong University, and Central South University, alongside collaborative efforts with Harvard University and France’s Institut National de la Santé et de la Recherche Médicale. The *H*-index has emerged as a widely accepted metric for assessing the scholarly productivity and impact level of researchers, effectively measuring the significance of their contributions as independent entities within their respective fields. To succinctly define the *H*-index, it represents the threshold such that at least H publications from a researcher have received a minimum of H citations each.^[35]^ The articles analyzed in this study reveal numerous researchers who have attained remarkable success in the domain of osteoporosis research. Notably, Robert R. Recker from Creighton University stands out as a prolific author, having engaged in extensive collaborations resulting in 636 of his works being indexed in the WOS database. Over the past 2 decades, his scholarly output has remained consistent, primarily focusing on osteoporosis, with an impressive total citation count of 36,161 and an H-index of 90. Similarly, Hongwen Deng from Tulane University is recognized as a significant contributor, with 796 coauthored publications recorded in the WOS, predominantly addressing endocrine diseases and osteoporosis. His work has garnered a total of 17,229 citations and he holds an H-index of 64. Furthermore, several other distinguished researchers with H-indices exceeding 50 are present, including Emma Frew from the University of Birmingham, who is a leading authority in osteoporosis studies. Most of these prominent authors are affiliated with prestigious institutions located in China, Europe, and the United States, fostering a collaborative research environment among them. Nevertheless, the data indicates an uneven global distribution of research pertaining to the omics of osteoporosis.

### 4.2. Knowledge map and analysis of research hotspots in osteoporosis omic research

Our analysis indicated that the majority of the articles reviewed in this study focused on osteoporosis-related transcriptomics and proteomics, with metabolomics studies following suit. Notably, transcriptomics and proteomics research has been ongoing from 2000 to 2024, while there has been a steady increase in metabolomics investigations since 2015. Furthermore, the pioneering DNA sequencing technique was developed by Frederic Sanger and his team in 1977, marking a significant advancement that paved the way for novel research avenues in the field of molecular biology.^[36]^ By the mid-1990s, microarray technology and gene chip technology had developed rapidly, and transcriptomics gradually became a hot spot for scientific research. Subsequently, the deciphering of the human whole genome sequence and the launch of functional omic research further promoted the development of life sciences. With these advances, scientists are increasingly concerned about how to carry out proteomics research using the model of genomic research in order to understand the complexity of biological systems more comprehensively.

From 2000 to 2004, the nascent stage of osteoporosis omic research, transcriptome technology began to be applied to the field of osteoporosis at an early stage, and although there were few publications, there were still several high-quality representative works, suggesting that the advanced microarray technology at that time was a powerful tool to reveal the etiological mechanisms of osteoporosis.^[13]^ More attention has been paid to the discovery of variants in osteoporosis-associated genes, and in a 2002 study by Arko, B, the authors were among the first to apply sequencing technology to screen for sequence variants in the promoter of the osteoprotectin gene and to analyze the correlation between the variants and postmenopausal osteoporosis.^[37]^ In the same year Prie, D sequenced mutations in the gene encoding the renal sodium phosphate cotransporter protein 2a, suggesting that heterozygous mutations in the phosphate cotransporter protein 2a gene may be responsible for hypophosphatemia and urinary phosphate loss in patients with osteoporosis.^[38]^ In addition, single nucleotide polymorphisms associated with osteoporosis were identified in the transforming growth factor TGF-β1, COL1A1 gene.^[39,40]^

Between the years 2005 and 2010, a gradual transition occurred, characterized by minimal fluctuations in the annual totals of both published articles and citations. Next-generation sequencing technology was introduced in 2005, with the launch of an ultra-high-throughput genome sequencing system based on pyrophosphate sequencing (the Roche 454 sequencing system), which enabled high-throughput, low-cost sequencing for osteoporosis diseases.^[41]^ Liu, the first in vivo microarray study of human osteoporosis proposes a new pathophysiological mechanism for osteoporosis, with increased recruitment of peripheral monocytes to bone and a greater tendency for monocytes to differentiate into osteoclasts.^[42]^ Later, Benisch, P suggested the need for sequencing and analyzing bone marrow mesenchymal stem cells from patients with osteoporosis, shifting the attention of scholars from the whole to a particular cell population.^[43]^ Genome-wide association studies (GWAS) serve as a powerful tool for identifying osteoporosis-associated single nucleotide polymorphisms,^[44]^ and a widely cited 2010 review article affirmed the need for previous work on the identification of osteoporosis variant genes and looked ahead to the potential of GWAS to uncover genetic variants in osteoporosis.^[45–47]^ Due to the phenotypic heterogeneity of osteoporosis diseases, subsequent efforts have continued to identify genetic variation in osteoporosis in different populations of humans,^[48]^ but due to the complexity of the human genome and the uncertainty of the etiology of the disease,^[49]^ GWAS has not yet been able to provide a single satisfactory answer to these questions.^[50]^ During this period, proteomics technology was also applied more frequently, Fan, YG applied proteomics technology earlier to detect differential proteins in estrogen-deficient rats, which provided valuable experimental evidence for elucidating the molecular mechanism of osteoporosis associated with estrogen loss.^[51]^

Between 2011 and 2019, overall research is in a precipitous phase, with advances in sequencing technology providing avenues for the discovery of new osteoporosis markers, and epigenomic technologies are being progressively applied during this phase.^[52]^ Previously, no scholars have systematically analyzed lncRNA, mRNA, circRNA, and miRNA expression profiles in osteoporosis patients,^[53,54]^ a large number of non-coding RNA molecules such as miR-194-5p and miR-422 a have been successively identified as potential miRNA biomarkers.^[55,56]^ Emerging disciplines such as genomics and bioinformatics have led to innovative bioinformatics approaches that can help researchers systematically analyze and interpret gene sequences, protein structures, gene expression, and metabolic pathways to delve into various aspects of osteoporosis.^[57,58]^ Osteoporosis is not just a single condition, but is closely related to the overall condition of an individual. The diversity of gut flora has been emphasized by a wide range of scholars during this period, and Wang, JH earlier conducted 16s ribosomal sequencing of gut microorganisms in osteoporosis patients to further understand the interactions between gut flora and bone health.^[59]^ Delgado-Calle linked osteoporosis to osteoarthritis by analyzing genome-wide methylation levels between the 2, with differentially methylated regions enriched in genes associated with cell differentiation and skeletal embryogenesis, leading to susceptibility to both diseases.^[60]^ In addition, metabolomics technology became a powerful tool for researchers to study the physiopathologic changes in osteoporosis during this period,^[61]^ and a study by Shum, LC focused attention on oxidative stress and mitochondria, where mitochondrial dysfunction and osteoclast senescence were strongly associated with bone loss.^[62]^ Beyond energy metabolism, the significance of variations in bone lipid metabolism levels in the development of osteoporosis is increasingly being investigated.^[63,64]^

The number of articles surged after 2019 and peaked in 2023 (Fig. 1), resulting in a low total number of citations for literature published between 2019 and 2024 due to the close proximity to the present day.The start of the neo-crown pneumonia epidemic in 2019 did not impede the progress of omic research; instead, the number of studies within the field grew at a faster rate from 2019 onwards, and the worldwide viral The outbreak triggered a growth in researchers’ interest in exploring the application of omic research within various fields, and scientists have applied omic techniques to the study of the neocoronavirus pathogen, with 13 viral susceptibility loci suggested in a whole-genome sequencing study of neocoronavirus-infected individuals from 19 countries.^[65]^ These studies are driving advances in omic research, and the working model of international collaborations emphasizes the possibility of discovering key genes in a wide range of complex human diseases. Notably, before 2019, few authors combined multiple omic approaches in their studies, whereas after 2019, researchers tend to explore disease mechanisms by combining multiple approaches, and the application of multiple omic studies provides new perspectives for understanding the complexity of osteoporosis diseases.^[66–69]^ Since the introduction of single-cell sequencing in 2010, this technology has provided an additional dimension to the understanding of genetic information, revealing the cellular composition of biological tissues and the regulatory relationships between genes.^[70]^ Bone has the specificity of osteoblasts and osteoclasts at different sites, and the heterogeneity of different populations of osteoblasts should be fully taken into account in the study of osteoporosis. Single-cell sequencing technology has opened the prelude to the study of the role of different populations of cells in osteoporosis, and the molecular dialog between the heterogeneity of the cellular populations in the skeletal muscle and the other populations of cells in the microenvironment has become an important pathway for the study of the disease mechanism of osteoporosis.^[68,71,72]^

### 4.3. Summary and outlook for future research

Twenty years ago, the collaboration of scientists from the United States, the United Kingdom, Japan, France, Germany and China completed the sequence map of the human genome, an achievement that has been hailed as a milestone in the history of life sciences. However, the completion of the Human Genome Project does not represent the end of genome research, but rather the opening of new research directions. Despite the increasing popularity of genetic technology, the question of the origin of life remains an unsolved mystery for scientists. For example, the questions of why the same fertilized egg develops into different tissue cells, and the order of gene expression in some relation to space and time, still plague the scientific community. These challenging questions will continue to drive scientists to delve deeper in order to unravel the mysteries of life.

The application of omic techniques within the field of osteoporosis has evolved at an astonishing rate since the 21st century, going through 3 main phases. In the first of these phases, research focused on performing omic studies on large numbers of mixed cells. Although this approach was able to obtain average gene expression levels of cell populations and was instrumental in advancing the understanding of the overall characterization of cell populations. However, it fails to provide detailed information on the gene expression of specific cells within the population.^[73,74]^ The second phase of research focused on sequencing single cells, an approach that allowed the study of gene expression to go down to the single-cell level, which in turn led to in-depth characterization of cell types and revealed cellular heterogeneity.^[72,75]^ However, gene expression is temporally and spatially specific. For temporal specificity, researchers can usually resolve it by performing single-cell transcriptome sequencing on samples from different time points. Performing single-cell studies requires enzymatic digestion of tissues to obtain cell suspensions, which results in the loss of information about the spatial location of tissues, and these are challenges with current technologies. High-throughput spatial transcriptomic technologies developed in recent years, such as 10x Genomics Visium spatial omic^[76]^ and GeoMx DSP spatial omic,^[77]^ have propelled the research into the third phase, providing new perspectives and tools for biological research. These techniques have made it possible to obtain gene expression profiling and spatial distribution data in tissue in situ, further advancing in-depth studies of real gene expression in tissue in situ cells. By combining single-cell and spatial transcriptomic technologies, researchers are able to integrate gene expression studies with the single-cell level, bringing new application prospects to the field of osteoporosis. This combined application provides a more comprehensive and in-depth understanding and approach to understanding the pathogenesis, diagnosis and treatment of osteoporosis. Recent studies have introduced live-cell transcriptome sequencing, a breakthrough technology that enables transcriptome sequencing of single cells while maintaining cell viability. With its full gene expression resolution and dynamic resolution, it brings a new breakthrough to the field of single-cell transcriptomics. This technology provides scientists with a unique means to directly and dynamically measure the transcriptome of a single cell while linking the cellular state to subsequent phenotypes, thus providing a more comprehensive and in-depth view of biological research.^[78]^

This study acknowledges several limitations. Firstly, data collection was exclusively conducted using the WOS database. Despite multiple adjustments to the search parameters, it was unavoidable that certain articles remained inaccessible. Secondly, the process of citation analysis can be subject to citation bias. This phenomenon occurs when older publications are cited more frequently by researchers, leading subsequent authors to reference articles that have previously garnered a high citation count. Consequently, the total citation figures may not accurately represent the actual content and quality of the publications. Such bias can particularly result in the underappreciation of significant research, especially that which is newly published, non-English-language, or originating from developing nations. Given that all the data utilized in this investigation are sourced from the WOSCC database, and that citation counts serve as one of the indicators of impact, these intrinsic biases could potentially influence the outcomes related to the identification of research hotspots and frontiers. Although scholars have put a lot of effort into exploring the path of osteoporosis mechanisms and have achieved some remarkable results, there is still much room for exploration. It is necessary for us to keep an eye on the research results related to osteoporosis in the next few years and keep abreast of the latest developments.

## 5. Conclusion

Overall, we have collected as comprehensively as possible the publications related to osteoporosis omic research at the beginning of the 21st century.To our knowledge, this study is the first comprehensive and objective bibliometric evaluation of the results of osteoporosis omic research, including international collaborations, research hotspots in previous years, and research frontiers, and these findings provide clues and ideas for scientific understanding of the development of this field and for solving the major current problems. The contribution of the United States in this field has been leading until 2014, and there have been rapid advances and major leaps in research in some Asian countries in the last decade. In the domain of osteoporosis genomics research, numerous studies encompassing transcriptomics, proteomics, and metabolomics have been conducted. Recently, advancements in technologies such as single-cell genomics and spatial genomics have begun to emerge. Looking ahead, it is anticipated that future investigations will increasingly favor collaborative efforts across institutions and nations, integrating various genomic approaches to elucidate the complexities of osteoporosis on a systemic scale.

## Author contributions

**Conceptualization:** Tao Bao.

**Data curation:** Xuyong Gong.

**Formal analysis:** Xuyong Gong.

**Writing – original draft:** Yuxia Yang.

**Writing – review & editing:** Tao Bao.
